# Effects of lignin and surfactant on adsorption and hydrolysis of cellulases on cellulose

**DOI:** 10.1186/s13068-016-0434-0

**Published:** 2016-01-26

**Authors:** Yanfei Li, Zongping Sun, Xiaoyan Ge, Junhua Zhang

**Affiliations:** College of Forestry, Northwest A and F University, 3 Taicheng Road, Yangling, 712100 China

**Keywords:** Cellulases, Lignin, Adsorption, Desorption, Tween 80, Enzymatic hydrolysis

## Abstract

**Background:**

Considerable works have been reported concerning the obstruction of enzymatic hydrolysis efficiency by lignin. However, there is a lack of information about the influence of lignin on the adsorption of cellulases on cellulose, along with the hydrolytic activity of the cellulases adsorbed on lignin. In addition, limited discovery has been reported about the influence of additives on cellulase desorption from lignin and lignocellulosic materials. In this work, the effects of lignin on cellulase adsorption and hydrolysis of Avicel were investigated and the effects of Tween 80 on cellulases adsorption and desorption on/from lignin and corn stover were explored.

**Results:**

The results showed that the maximum adsorption capacity of Avicel reduced from 276.9 to 179.7 and 112.1 mg/g cellulose with the addition of 1 and 10 mg lignin per gram Avicel, which indicated that lignin adsorbed on Avicel reduced surface area of cellulose and lignin available for cellulases. Cellulases adsorbed on lignin could be released by reaching new adsorption equilibrium between lignin and supernatants. In addition, cellulases desorbed from lignin still possess hydrolytic capacity. Tween 80 could adsorb onto both lignin and corn stover, and reduce the cellulase adsorption on them. Furthermore, Tween 80 could enhance desorption of cellulases from both lignin and corn stover, which might be due to the competitive adsorption between cellulases and Tween 80 on them.

**Conclusions:**

The presence of lignin decreased the maximum adsorption capacity of cellulases on cellulose and the cellulases adsorbed on lignin could be released to supernatant, exhibiting hydrolytic activity. Tween 80 could alleviate the adsorption of cellulases and enhanced desorption of cellulases on/from lignin and corn stover. The conclusions of this work help us further understanding the role of lignin in the reduction of adsorption of cellulases on substrates, and the function of additives in cellulases adsorption and desorption on/from lignin and substrates.

## Background

Agricultural residues are abundant, cheap, and sustainable materials for the production of bio-ethanol. One of the bottlenecks of its commercialization is the high costs and low efficiency of enzymes required for hydrolysis of cellulosic materials into fermentable sugars [1]. In order to efficiently convert biomass into sugars, it should maximally remove lignin and minimally modify polysaccharide by pretreatment [2]. However, part of lignin inevitably existed in the pretreated materials in some pretreatment processes, which has been shown to affect both enzymatic hydrolysis and enzyme recycling [3, 4].

In the past three decades, the negative effects of lignin isolated from nature biomass in the cellulose hydrolysis have been wildly studied [5–9]. The inhibition mechanisms can be attributed to the enzyme binding with lignin and physical blocking of enzymes by lignin. The cellulases have been proposed to be adsorbed on lignin via hydrophobic interactions, electrostatic, and hydrogen bonding interactions [6, 7]. In the unspecific binding of cellulases to lignin, the binding efficiency markedly decreased by removing of carbohydrate-binding module (CBM) from cellulases or mutating aromatic and polar residues on the planar face of the CBM [10, 11]. The intensity of adsorption interaction depends on the variety of enzyme and lignin. It has been reported that the cellulases are more strongly inhibited by two lignins than the two xylanases or the β-glucosidase preparation [12]. In addition, the adsorption characteristics of different β-glucosidase preparations are different [13]. Lignin isolated from herbaceous plants is less inhibiting to enzymes than that from wood [14], and lignin isolated from non-pretreated raw biomass exhibited less inhibiting effects on enzymes than lignin isolated from pretreated biomass, such as by steam explosion [15]. To reduce the recalcitrance of lignin, many methods have developed to maximize lignin removal. However, pretreatment methods aiming at lignin removal are usually expensive and some-times raise environmental concerns. Alternatively, genetically modified energy plants that have reduced lignin content would meet the demand [16]. Considerable works have been reported concerning the obstruction of enzymatic hydrolysis efficiency by lignin. However, there is a lack of information about the influence of lignin on the adsorption of cellulases on cellulose, along with the hydrolytic capacity of the cellulases adsorbed on lignin.

The application of additives, such as non-ionic surfactants [17] and polymers [18] is an efficient way to reduce the non-productive adsorption of enzymes onto lignin in the hydrolysis of biomass. The principle has been discovered is the additives could adsorb on the exposed lignin to prevent unspecific adsorption of cellulases, thereby producing better recycles of enzymes and higher hydrolysis yields [19, 20]. Park et al. [21] examined the effect of several surfactants on enzymatic hydrolysis of newspaper, and found Tweens to be among the best performers, with two times higher conversion at 80 h than that without surfactant. Qing et al. also observed that Tween 80 reduced non-productive binding of enzymes on the biomass surface and increased enzymatic hydrolysis yields more than dodecylbenzene sulfonic acid and polyethylene glycol 4000 [22]. In previous study, Tween 80 had the higher effect on the xylanase adsorption and desorption on/from lignin than PEG [23]. However, limited discovery has been reported about the influence of additives on hydrolytic capacities of cellulase desorbed from lignin and lignocellulosic materials.

In this work, the impact of lignin on adsorption of cellulases on Avicel was investigated, and desorption of cellulases from lignin and their hydrolytic activities were explored. Furthermore, the effects of Tween 80 on cellulases adsorption and desorption onto/from lignin and dilute-acid pretreated corn stover were investigated. The objective of this work is to explore the influence of the extraneous lignin on cellulase adsorption on cellulose and the role of additives in impediment of adsorption of cellulases on lignocelluloses.

## Results and discussion

### Effect of lignin on cellulose hydrolysis

**Fig. 1:**
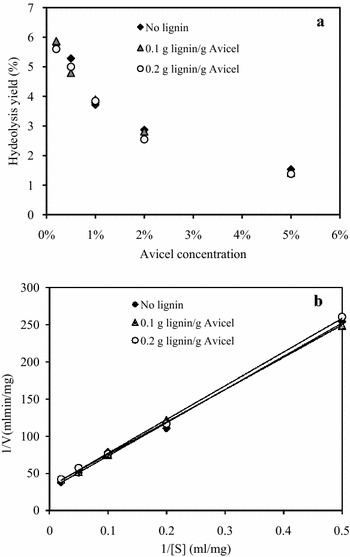
Effect of lignin on the initial hydrolysis rate of CEL. The hydrolysis of different concentrations of Avicel by CEL (0.68 mg Celluclast 1.5L supplement with 0.33 mg Novozyme 188) was performed at 50 °C for 30 min hydrolysis (**a**). Relationship between 1/[S] and 1/[V] within 30 min (**b**)

The inhibition of lignin on enzymatic hydrolysis of lignocellulosic materials has been confirmed by many authors [5–7]. However, there is a lack of information on the effect of lignin on the initial hydrolysis rate of cellulose. In this work, the hydrolysis yields of Avicel by the combination of Celluclast 1.5L and Novozyme 188 (CEL) in 30 min with different dosages of lignin were determined. With the increase of Avicel consistency from 0.2 to 5.0 %, the hydrolysis yield of Avicel dwindled rapidly from 5.7 to 1.5 % (Fig. 1a), which was due to the lower CEL dosage per gram Avicel at higher consistency. However, with the increase of lignin consistency, the hydrolysis yields of Avicel not clearly decreased. It is known that the different components of CEL (endoglucanase, cellobiohydrolase, and β-glucosidase) exhibit different adsorption behaviors with lignin [6, 12, 13], and the presence of lignin change the ratio of enzyme activities in supernatant needed for synergism during cellulose hydrolysis. The results here indicated that the enzyme components were abundant for the hydrolysis and slightly influenced by lignin in the initial 30 min, which was in good agreement with the report that the enzyme adsorbed onto non-cellulosic component of a pretreated feedstock is not likely to have a major impact on cellulose hydrolysis [24].

The results of kinetic analysis in the presence of different concentrations of lignin were presented as Lineweaver–Burk plots of 1/V vs. 1/S (Fig. 1b). Based on the intersection of the lines neither on the Y-axis nor X-axis, the inhibitory effect of lignin on CEL was not competitive or non-competitive. Considering these results, it seems likely because of the adsorption interaction not only between lignin and cellulases, but also between lignin and cellulose. Sewalt et al. [25] reported that the interaction between lignin and carbohydrate/enzyme occurred after the addition of extraneous lignins, which supported the results in this work.

### Effect of lignin on CEL adsorption on cellulose

**Fig. 2:**
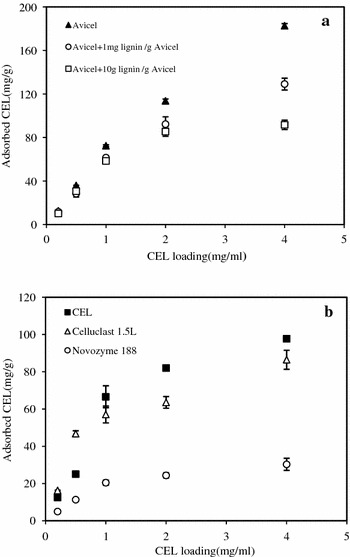
Adsorption kinetics of cellulases on Avicel and lignin. Effect of lignin on the adsorption kinetics of CEL on Avicel (**a**). Adsorption kinetics of CEL, Celluclast 1.5 L, and Novozyme 188 on 1 % lignin (**b**). The adsorption experiments were performed using CEL (Celluclast 1.5L and Novozyme 188 at a FPU: nkat ratio of 1:50) in 50 mM sodium citrate buffer at 4 °C for 60 min. *Error bars* represent standard errors

To investigate the effect of lignin on the adsorption of cellulases to cellulose, the adsorption kinetics of CEL on Avicel (with and without acid-insoluble lignin) as well as CEL on lignin were carried out. Adsorption isotherms of CEL on Avicel were found to follow the Langmuir equation very well with *R*^*2*^ >0.90 (Fig. 2a, Table 1). The results revealed that after the addition of 1 and 10 mg lignin/g Avicel, the maximum adsorption capacity of Avicel (P_ads_, _m_ = 276.9 mg/g Avicel) decreased to 179.7 and 112.1 mg/g Avicel, respectively. The results here demonstrated that the addition of lignin induced the less maximum adsorption capacity of CEL on Avicel (Table 1). If there is no adsorption between Avicel and lignin, the maximum adsorption capacity of cellulases on Avicel and lignin should exceed that of cellulases on Avicel (276.9 mg/g). However, the maximum adsorption capacities decreased to 180–112 mg/g after the addition of lignin. Thus, the explanation was that the lignin adsorbed onto Avicel sequentially reduced the surface area of cellulose and lignin available for CEL [7]. Selig et al. [26] also reported that the lignin droplets produced from corn stover during pretreatment under acidic pH can deposit back onto the surface of residual biomass.

**Table 1:** Adsorption parameters of cellulase and maximum adsorption capacities of cellulose for Avicel with lignin

Substrate	Maximum adsorption capacity P_ads, m_ (mg/g)	Affinity constants K_p_ (ml/mg)	Strength of binding, A (ml/mg)	*R* ^*2*^
Avicel	276.9	0.9	246.7	0.90
Avicel + 1 mg lignin/g Avicel	179.7	1.0	174.4	0.98
Avicel + 10 mg lignin/g Avicel	112.1	1.7	192.0	0.96
Lignin	118.1	1.7	199.7	0.96

Experiments were performed in triplicates and the mean values are presented

The adsorption isotherms of CEL, Celluclast 1.5L, and Novozyme 188 on acid-insoluble lignin followed the Langmuir equation very well, as evident by statistical correlation coefficients *R*^*2*^ >0.9 (Fig. 2b, Table 2). Celluclast 1.5L had higher maximum adsorption capacity than Novozyme 188, which could be due to the absence of CBM in β-glucosidase of Novozyme 188 since CBM plays an important role in adsorption on lignin [6]. Pareek et al. [6] noticed that the maximum adsorption capacity of Celluclast 1.5L was higher than that of Novozyme 188 on acid-insoluble spruce lignin, which was in good agreement with the data in this work (Table 2).

**Table 2:** Adsorption parameters of cellulases for lignin

	Maximum adsorption capacity P_ads, m_ (mg/g)	Affinity constants K_p_ (L/g)	Strength of binding, A (ml/mg)	*R* ^*2*^
CEL	118.1	1.7	199.7	0.96
Celluclast 1.5L	88.1	5.0	436.1	0.98
Novozyme 188	37.9	1.2	45.1	0.99

It is generally believed that CEL could adsorb on cellulose by the interactions between CBM of the enzyme with the two hydrophobic crystal faces of cellulose [27]. While the adsorptions of cellulases onto lignin are mainly due to the hydrophobic interactions, electrostatic interactions, etc. [6, 7]. It is well known that the isolation method of lignin affects the properties of lignin and consequently results in different adsorption capacity toward lignin. In this work, the structure of lignin prepared by two-step acid hydrolysis could be different from the native lignin in lignocelluloses and exposed lignin in pretreated biomass. However, the lignin in pretreated substrates could also adsorb cellulases and affect the cellulase adsorption and hydrolysis toward cellulose. Therefore, the data here would provide scientific guidance for the adsorption behavior of cellulases on pretreated substrates, especially dilute-acid pretreated lignocellulosic materials, in which the lignin is mostly exposed.

### Desorption of cellulases from lignin

Since the non-productive adsorption of cellulolytic enzymes on lignin is unavoidable, the adsorption and desorption behavior of cellulases on/from lignin, and the hydrolytic activity of cellulases released from lignin should be paid attention. It was noticed that 56.4 % of CEL protein was adsorbed on lignin, and 10.3 % of CEL protein was released from lignin by first time washing with buffer (Table 3). Compared to native lignin or lignin prepared by liquid hot water pretreatment, lignin isolated by two-step sulfuric acid treatment, causing condensation reactions, might contain more phenolic hydroxyl groups and less aliphatic hydroxyls, and exhibit higher affinity and adsorption capacity toward cellulases [6, 28]. After three times of washing with buffer, the released CEL protein reached 20.5 %, indicating about 36 % of CEL protein was still adsorbed on lignin. Desorption of CEL from lignin at 4 °C and 50 °C by buffer were compared and the results showed that more CEL protein was released at 50 °C (15.4 %) than that at 4 °C (5.1 %). The temperature of 50 °C was chosen because it is the optimal hydrolysis temperature for commercial cellulase preparations. The results here confirmed that cellulases adsorbed on lignin could be released by reaching new adsorption equilibrium between lignin and supernatants, with different amounts of CEL protein and different temperatures.

**Table 3:** Amounts of protein of desorbed CEL from lignin-CEL complex by washing with fresh buffer

	Protein in supernatant (mg/ml)	Percent of total protein (%)	Hydrolysis yield^c^ (%)
Loading	0.39	100	nd^d^
Adsorption supernatant	0.17	43.6	5.9
First supernatant^a^	0.04	10.3	4.0
Second supernatant	0.02	5.1	1.6
Third supernatant	0.02	5.1	1.2
Desorption at 50 °C^b^	0.06	15.4	4.0
Desorption at 4 °C^b^	0.02	5.1	nd^d^

^a^The first to third supernatant obtained from the solid washed with 2 ml buffer at room temperature

^b^The desorption supernatant obtained from the lignin-CEL complex incubated with 2 ml buffer at 50 or 4 °C for 10 min

^c^The hydrolysis of 1 % Avicel was performed at 50 °C for 48 h with a working volume of 3 ml

^d^Not determined

The hydrolysis yields of Avicel by supernatants from three times buffer washing was observed. Moreover, the hydrolysis yield of Avicel by CEL desorbed from lignin by buffer at 50 °C was 4.0 %, indicating that the released CEL still exhibited hydrolytic activity. Similar observations have been reported that cellulases desorbed from lignocresol-cellulase complex and cellulosic materials are active [29, 30], which was consistent with the results here.

### Enzymatic activity of lignin-cellulase complex

**Fig. 3:**
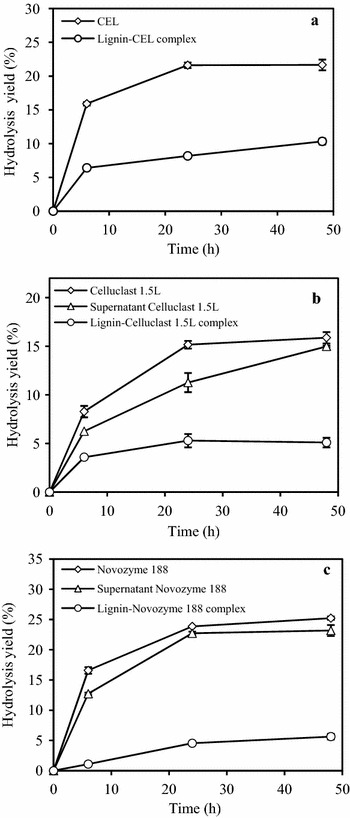
Hydrolytic capacities of different lignin-enzyme complex. Hydrolysis of 1 % Avicel and 1 % cellobiose by lignin-CEL complex (**a**), lignin-Celluclast 1.5L complex (**b**) and lignin-Novozyme 188complex (**c**) in 50 mM sodium citrate buffer at pH 5.0 for 48 h. CEL composed of Celluclast 1.5L (22.7 mg/g lignin) and Novozyme 188 (11.1 mg/g lignin). The doses of Celluclast 1.5L and Novozyme 188 were 22.7 and 11.1 mg/g lignin, respectively. *Error bars* represent standard errors

The lignin was incubated with CEL at 4 °C for 30 min and was washed three times with sodium citrate buffer at room temperature, which was used as lignin-cellulase complex for following hydrolysis experiments. The “supernatant” cellulases were the supernatant from cellulases and lignin system after centrifugation. As shown in Fig. 3a, the hydrolysis yields of Avicel by CEL and lignin-CEL complex in 48 h were 21.7 and 10.3 %, respectively. Hydrolysis yields of 1 % Avicel and cellobiose by lignin-Celluclast 1.5L complex and lignin-Novozyme 188 complex in 48 h were 5.1 and 5.6 %, respectively (Fig. 3b, c). The results indicated that both Celluclast 1.5L and Novozyme 188 adsorbed on lignin could exhibit hydrolytic capacity. The hydrolytic capacity of lignin-enzyme complex could partly be explained by the released enzyme from lignin exhibiting activity in the hydrolysis process, as confirmed in Table 3. Another explanation for the activity of lignin-CEL complex likely attributed to that the CEL bound to lignin could be functioned as an immobilized CEL in hydrolysis process [31]. Obviously, the hydrolysis yields of Avicel by CEL were higher than those by lignin-CEL complex, owing to higher amount of cellulases and the absence of lignin in CEL. In addition, as shown in Table 2 and Fig. 2b, the adsorption of β-glucosidase on lignin was much lower than Celluclast 1.5L, which meant that more β-glucosidase was washed away by the buffer after CEL adsorption. Hence, the hydrolytic capacity of lignin-CEL complex was lower than that of CEL since the synergism between β-glucosidase and other cellulase components is of great importance for cellulose hydrolysis.

### Effect of Tween 80 on adsorption of CEL on lignin and corn stover

**Fig. 4:**
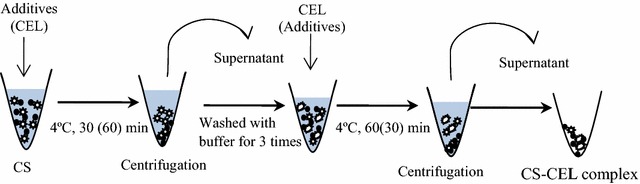
Flow diagram of the preparation of CS-CEL complex. ● CS,  additives,  CEL

**Fig. 5:**
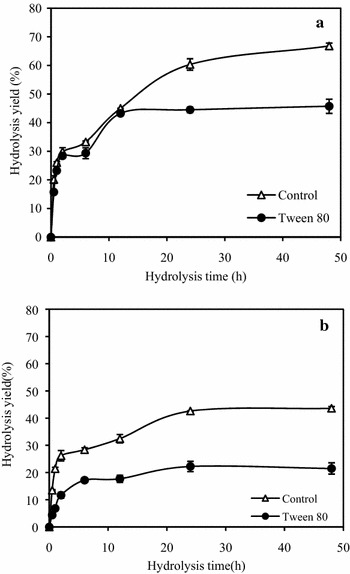
Hydrolysis of 1 % CS by CEL desorbed from CS-CEL complex.** a** The CS-CEL complex was obtained from incubating CS with Tween 80 (2.5 mg/ml) or buffer followed by CEL (100 mg/g CS).** b** The CS-CEL complex was obtained from incubating CS with CEL (100 mg/g CS) followed by Tween 80 (2.5 mg/ml) or buffer. The hydrolysis was performed in 50 mM sodium citrate buffer (pH5.0) at 50 °C for 48 h. *Error bars* represent the standard errors

The effect of additives on the adsorption of CEL onto lignin was unclear and investigated here by examining the hydrolysis yields of Avicel by lignin-CEL complex obtained from adsorption experiments with and without Tween 80. The results showed that the hydrolysis yields of Avicel by lignin-CEL complex decreased from 9.8 to 4.4 % with Tween 80. The results indicated that Tween 80 reduced the CEL adsorption onto lignin by strong adsorption of Tween 80 onto lignin. Furthermore, the effect of Tween 80 on adsorption of CEL onto dilute-acid pretreated corn stover (CS) was tested by the hydrolysis of CS by CS-CEL complex obtained from incubating CS with Tween 80 or buffer followed by CEL (Fig. 4). Dilute-acid pretreated corn stover was used as the substrate because of its high lignin content (31.6 %) and the presence of lignin played an important role in the adsorption of cellulases on substrates. As shown in Fig. 5a, the hydrolysis yield of CS by the CS-CEL complex without Tween 80 reached 66.8 %, which was much higher than that (45.7 %) by CS-CEL complex with Tween 80. The reduction of hydrolysis yield with Tween 80 was proposed to be partly attributed to the reduction of CEL adsorption on lignin in CS by Tween 80 and less amount of CEL released from the CS-CEL complex. Consistent with the findings here, another study showed that the adsorption of cellulase on cellulose with Tween 80 addition decreased about 15 % [32]. Similar observations have been reported with Tween 20, which adsorbed onto lignocelluloses and affect the interaction between enzyme and lignocelluloses [33].

### Effect of Tween 80 on desorption of CEL from lignin and corn stover

The role of Tween 80 in CEL desorption from acid-insoluble lignin was also investigated and the results showed that with the presence of Tween 80, the hydrolysis yield of Avicel by lignin-CEL complex decreased by 27.6 %. Such decrease in hydrolysis yield was due to less CEL available for hydrolysis by desorption of CEL from lignin, which might be due to the competitive adsorption between CEL and Tween 80 on lignin. Thus, the addition of Tween 80 both before and after cellulases could be beneficial for the hydrolysis of lignocellulosic materials. The competitive adsorption between non-ionic surfactant Tween 80 and cellulases on lignin suggested the surfactant with higher affinity with lignin would be more beneficial for the reduction of cellulase adsorption and potentially reduce the dosages of surfactant and cellulases.

The effect of Tween 80 on desorption of CEL from CS was investigated as well. As represented in Fig. 5b, the hydrolysis yield (43.6 %) of CS by CS-CEL complex without Tween 80 was higher than that (21.5 %) with Tween 80. The data demonstrated that more CEL was desorbed from CS with the present of Tween 80 by competitive adsorption between CEL and Tween 80 on lignin and cellulose in CS, and the lower amount of CEL available for hydrolysis. It has been observed by Park et al. [21] that surfactants help the cellulases to desorb from the binding sites on the surface of used newspaper. Zhang et al. [34] also reported the positive effects of PEG on cellulases desorption from steam-exploded corn stover, microcrystalline cellulose, and bagasse sulfite pulp.

In this work, the effects of non-ionic surfactant Tween 80 on cellulases adsorption and desorption on/from acid-insoluble lignin had been elucidated. In addition, the negative effects of acid-insoluble lignin on cellulase adsorption and hydrolysis of Avicel had been confirmed. It has been reported that the high adsorption affinity of cellulase to lignin could be partially due to the hydrophobic interactions between aromatic residues of cellulases on the flat surface of CBM such as tyrosine or tryptophan and the hydrophobic surfaces of lignin [35, 36]. However, the composition and structure in lignin contributed the interaction with cellulases were still in controversial [37]. Since the source of substrates, pretreatment method, and isolation method would affect the structure of lignin and consequently impact adsorption and desorption of cellulases on/from lignin, the influences of various lignin on those aspects (adsorption, desorption, and hydrolysis) of cellulases toward cellulose need to be further investigated.

## Conclusions

The presence of lignin decreased the maximum adsorption capacity of cellulases on cellulose, which could be due to the reduction of surface area of cellulose and lignin available for cellulases by adsorption of lignin on cellulose. The cellulases adsorbed on lignin could be released to supernatant, exhibiting hydrolytic activity. Tween 80 could alleviate the adsorption of cellulases on corn stover partly due to the adsorption of Tween 80 onto lignin, which occupied partial hydrophobic surface of lignin in corn stover. In addition, Tween 80 enhanced desorption of cellulases from lignin and corn stover because of competitive adsorption between cellulases and Tween 80 on lignin and corn stover. The conclusions of this work help us further understanding the role of lignin in the reduction of adsorption of cellulases on substrates, and the function of additives in cellulases adsorption and desorption on/from lignin and substrates.

## Methods

### Substrates

Corn stover was collected in Yangling, Shaanxi Province, China, and was milled and sieved through a 60 mesh screen scale (≤0.3 mm). The dilute-acid pretreated corn stover (CS) was pretreated with 1 % H_2_SO_4_ at a solid-to-liquid ratio of 1:10 and 121 °C for 1 h. The chemical compositions of CS before and after pretreatment were determined according to the Laboratory Analytical Procedures established by the National Renewable Energy Laboratory [38–40] and are shown in Table 4.

**Table 4:** Chemical compositions of corn stover and dilute-acid pretreated corn stover (% of dry matter)

Sample	Cellulose	Xylan	Acid-insoluble lignin	Acid-soluble lignin	Ash	Extractives
Corn stover	32.6 ± 0.2	20.5 ± 0.0	21.0 ± 0.1	3.4 ± 0.1	6.7 ± 0.1	12.4 ± 0.5
Dilute-acid pretreated corn stover ^a^	47.4 ± 2.3	7.2 ± 0.0	31.6 ± 1.5	1.5 ± 0.1	5.6 ± 0.1	10.9 ± 0.3

Data presented are mean values of two independent measurements

^a^Corn stover was pretreated with 1 % H_2_SO_4_ at a solid-to-liquid ratio of 1:10 and 121 °C for 1 h

Microcrystalline cellulose Avicel PH 101, and D-(+)-cellobiose were purchased from Sigma Chemical Co. (St. Louis, MO, USA). The contents of cellulose and xylan in Avicel were 91.3 and 1.23 %, respectively. In addition, Avicel contained some mannan (1.45 %), arabinan (<0.1 %), galactan (<0.1 %), and lignin (<0.1 %) [41]. Tween 80 was purchased from Tianjin Kermel Reagent Co. Ltd, China.

### Lignin preparation

The lignin from corn stover was isolated from the milled materials by a two-step sulfuric acid hydrolysis according to the method of National Renewable Energy Laboratory [38]. The lignin obtained by this method was acid-insoluble lignin, which was washed by deionized water to neutral and was lyophilized later to used as lignin preparation for following experiments.

### Enzymes

The two enzyme preparations used in the study were obtained from Novo Nordisk A/S, Bagsvaerd, Denmark. Celluclast 1.5L had an activity of 74.7 FPU/ml (169.6 mg protein/ml) measured according to International Union of Pure and Applied Chemistry standard assay [42]. The activity of Novozyme 188 was determined to be 8451 nkat/ml (187.9 mg protein/ml) of β-glucosidase as described by the Ref. [43].

### Adsorption kinetics

The experiments were carried out in 50 mM sodium citric acid buffer (pH 5.0) in 1 % consistency of Avicel or lignin with 2 ml volume. The samples were incubated with 10–400 mg protein of enzymes/g dry matter for 60 min at 4 °C with magnetic stirring. After adsorption the solids and liquids were separated by centrifugation (10,000×*g*, 10 min). The protein adsorbed was measured by subtracting the protein in supernatant from the total protein loaded. The enzyme preparations used for this adsorption experiments included Celluclast 1.5 L, Novozyme 188, and their combined cellulase preparation (CEL). To investigate the effect of lignin on adsorption kinetics of CEL on Avicel, different concentrations of lignin preparations (1 and 10 mg lignin/g Avicel) were added in the incubation system. All adsorption experiments were carried out in triplicates and average values are presented.

### Calculation of adsorption parameters

Adsorption parameters were calculated according to the Langmuir-type adsorption isotherm as the equation below [44].

$$ P_{\rm{ads}} = \frac{{K_ {\rm{p}} \cdot P_{\rm{ads, \,m}} }}{{1 + K_ {\rm{p}} \cdot P}} \cdot P $$

where *P*_ads, m_ is the maximum amount of adsorbed enzyme (mg enzyme/g solid); *P* is the amount of free enzyme (mg enzyme/ml) in supernatant after adsorption; *K*_p_ is the adsorption equilibrium constant (ml/mg enzyme) and is a measurement for the adsorption affinity. *P*_ads, m_ and *K*_p_ can be calculated from the plots of *P*/*P*_ads, m_ vs. *P*, and the adsorption strength of enzyme (*A*) is calculated from *P*_ads, m_ and *K*_p_ (*A* = *P*_ads, m_ ×  *K*_p_).

### Desorption of cellulases from lignin

The incubation of CEL (22.7 mg Celluclast 1.5L/g lignin and 11.1 mg Novozyme 188/g lignin) with lignin was carried out in 50 mM sodium citric acid buffer (pH 5.0) with 1 % lignin at a working volume of 2 ml, which was performed at with magnetic stirring for 60 min. After adsorption the solid and liquid were separated and the solid was washed three times with 2 ml buffer. The washed solid was further incubated with 2 ml buffer at 4 and 50 °C for 10 min, respectively. All supernatants after buffer washing and incubation were used for Avicel (1 %) hydrolysis at 50 °C for 48 h with a working volume of 3 ml.

### Preparation of lignin-enzyme complex

The incubation of CEL, Celluclast1.5 L, and Novozyme 188 with lignin was carried out in 50 mM sodium citric acid buffer (pH 5.0) with 1 % lignin at a working volume of 2 ml, which was performed at 4 °C with magnetic stirring for 30 min. After adsorption the solid and liquid were separated and the solid was washed three times with 4 ml buffer. The washed solid was used as lignin-enzyme complex following hydrolysis experiments. For comparison, the liquid obtained from centrifugation was also used for hydrolysis experiments.

### Preparation of CS-CEL complex

The adsorption experiment was conducted in a system of 50 mM sodium citrate buffer (pH 5.0) with 1 % (w/v) CS with a working volume of 1.2 ml. The system was incubated with 0 or 2.5 mg/ml Tween 80 at 4 °C with magnetic stirring for 30 min. The solid and liquid in the system were separated by centrifugation (10,000×*g*, 10 min), and the solid was washed with 5 ml buffer for three times. After that, the solid was incubated with CEL (1.0 mg/ml) in 1.2 ml buffer at 4 °C with magnetic stirring for 60 min, then the solid and liquid were separated and the solid was washed with 5 ml buffer for three times and was used as CS-CEL complex for hydrolysis experiments.

Desorption experiments were also carried out in a system as described above but CS was incubated with CEL followed by Tween 80. The flow diagram of the preparation of CS-CEL complex is shown in Fig. 4.

### Hydrolysis experiments

The hydrolysis of Avicel, cellobiose, and CS were performed in screw-capped 10 ml tubes containing 0.02 % NaN_3_ in 50 mM sodium citrate buffer (pH 5.0) at 50 °C using an orbital shaker with 200 rpm. The enzyme preparations used for hydrolysis experiments included CEL, lignin-enzyme complex, and CS-CEL complex. The effect of lignin on the initial hydrolysis rate of Avicel by CEL was investigated with lignin added at varying concentrations to the enzymatic Avicel hydrolysis reaction. Samples were withdrawn at 0.5–48 h and boiled for 10 min to stop the enzymatic hydrolysis. After cooling to room temperature, the samples were centrifuged and the supernatants were collected for further analysis. All experiments were performed in duplication and average values are presented.

### Analytical methods

Reducing sugars were measured using the dinitrosalicylic acid method with glucose as the standards [45]. The enzyme protein contents were determined by the standard BCA method [46]. Two replicate tests were carried out and average values are presented.

The hydrolysis yields of Avicel by CEL, lignin-enzyme complex, or CS by CS-CEL complex were calculated according to the following formula:

$$ {\text{Hydrolysis yield}} \, \left( \% \right) = \frac{{{\text{Reducing sugars released}}\times0.9}}{\text{Theoretical amount of cellulose and xylan in substrates}} \times 100 $$

The hydrolysis yields of cellobiose by lignin-Novozyme 188 complex were calculated according to the following formula:

$$ {\text{Hydrolysis yield }}\left( \% \right) = \frac{{{\text{Glucose released}}\times0.9}}{\text{Total amount of cellobiose}} \times 100 $$

## Abbreviations

A: strength of binding
CEL: cellulases
CS: dilute-acid pretreated corn stover
K_p_: affinity constants
P_ads, m_: adsorption capacities
CBM: carbohydrate-binding module
